# Unveiling the Catalytic Mechanism of Abl1 Kinase: A Single-Magnesium Ion Pathway for Phosphoryl Transfer

**DOI:** 10.1021/acs.biochem.4c00838

**Published:** 2025-03-05

**Authors:** Sinisa Bjelic, Stella Hernandez Maganhi, Ran Friedman

**Affiliations:** † Department of Chemistry and Biomedical Sciences, Linnaeus University, Stuvaregatan 4, 392 31 Kalmar, Sweden; ‡ Department of Exact and Earth Sciences, State University of Minas Gerais (UEMG), Vereador Geraldo Moisés Street, 38302-192 Ituiutaba, Minas Gerais, Brazil

## Abstract

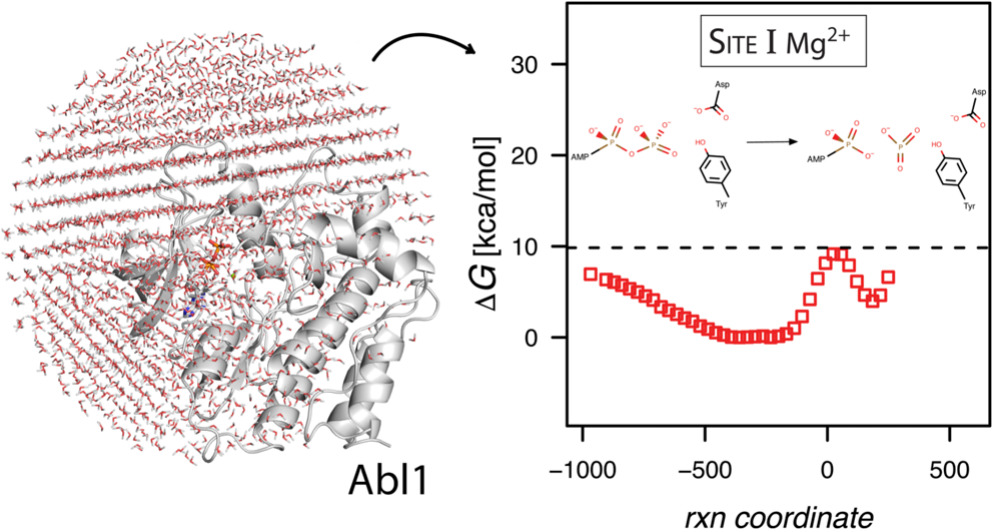

Abl1, a nonreceptor tyrosine kinase closely related to Src kinase, regulates critical cellular processes like proliferation, differentiation, cytoskeletal dynamics, and response to environmental cues through phosphorylation-driven activation. Dysregulation places it centrally in the oncogenic pathway leading to blood cancers. making it an ideal drug target for small molecule inhibitors. We sought to understand the underlying mechanism of the phosphoryl-transfer step from the ATP molecule to the substrate tyrosine, as carried out by the Abl1 enzyme. By calculating free energy profiles for the reaction using the empirical valence bond representation of the reacting fragments paired with molecular dynamics and free energy perturbation calculations, a combination of several plausible reaction pathways, ATP conformations, and the number of magnesium ion cofactors have been investigated. For the best-catalyzed pathway, which proceeds through a dissociative mechanism with a single magnesium ion situated in Site I, a close agreement was reached with the experimentally determined catalytic rates. We conclude that the catalytic mechanism in Abl1 requires one magnesium ion for efficient catalysis, unlike other kinases, where two ions are utilized. A better overall understanding of the phosphoryl-transfer reactions in Abl1 can be used for type-I inhibitor development and generally contributes to a comprehensive overview of the mechanism for ATP-driven reactions.

## Introduction

Abl1, a nonreceptor tyrosine kinase closely related to Src kinase, plays a crucial role in regulating a variety of cellular processes, including cell proliferation, differentiation, and response to environmental stressors.^1^ It is primarily activated by extracellular signals, such as those from growth factors, which leads to its phosphorylation on specific tyrosine residues. The phosphorylation results in conformational changes that activate Abl1, enabling it to interact with and phosphorylate a range of target proteins.^2^ The protein–protein network is vital for modulating the cytoskeletal dynamics of cells and facilitates processes such as migration and adhesion, which are critical for tissue development and repair. Additionally, Abl1 has been implicated in the regulation of gene expression and cell survival pathways, further underscoring its importance in maintaining cellular homeostasis. The dysregulation of Abl1 kinase activity is associated with several pathological conditions, most notably chronic myeloid leukemia (CML).^3−5^ In CML, the BCR–ABL fusion protein, resulting from a chromosomal translocation, exhibits constitutive kinase activity that drives uncontrolled cell proliferation and resistance to apoptosis. This abnormal activation of Abl1 disrupts normal signaling pathways, leading to the proliferation of myeloid progenitor cells. The clinical significance of Abl1 has prompted extensive research into its inhibition as a therapeutic strategy, with tyrosine kinase inhibitors (TKIs) such as imatinib demonstrating efficacy in treating CML by selectively targeting the BCR–ABL fusion protein.^6,7^

**Figure 1:**
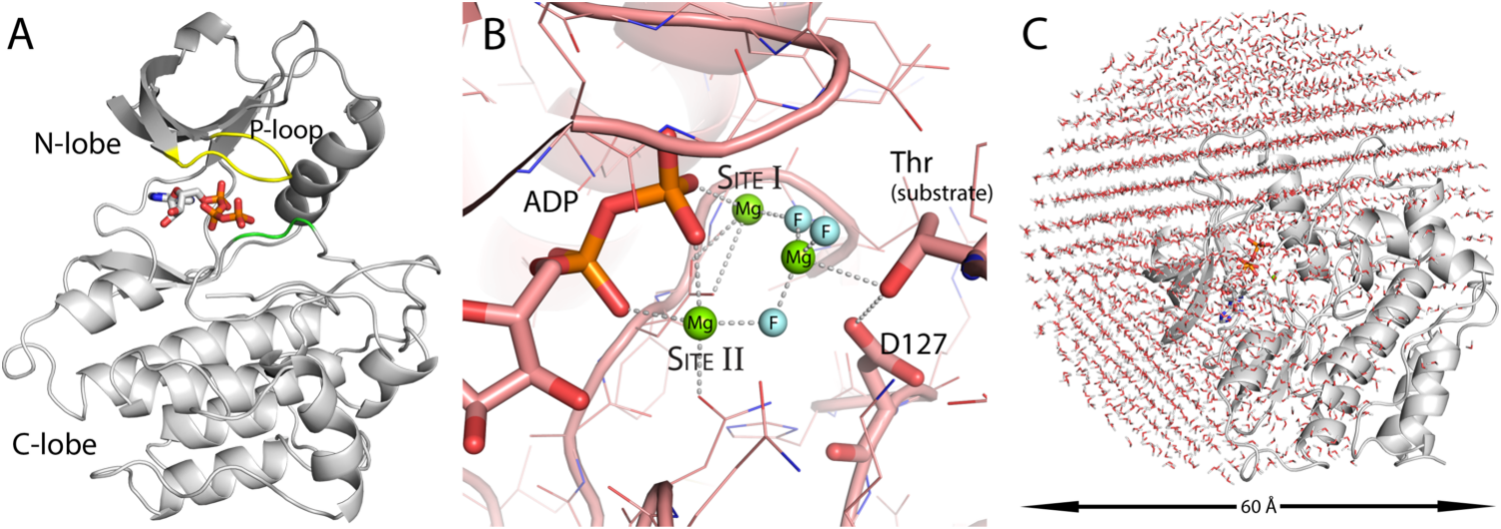
Abl1 structure and simulation setup. (A) The kinase domain, in cartoon representation, with the N-lobe colored dark gray and the C-lobe light gray. The P-loop, involved in phosphoryl binding, is shown in yellow; the DGF segment, that flips into the active conformation, is shown in green. (B) Definition of the magnesium ion binding sites in CDK2 kinase (PDB ID 3QHW). The green spheres are magnesium ions. Site I ion mainly interacts with the β phosphoryl group and the metaphosphate intermediate mimic (MgF_3_^–^). Site II magnesium ion interacts with both α and β phosphoryl groups, as well as the intermediate mimic. (C) The system used for the molecular dynamics simulations consisted of the Abl1 kinase domain solvated with a 60 Å water sphere centered on the phosphorus γ atom of the ATP molecule.

Understanding the precise mechanisms of Abl1 function and its regulatory pathways is essential for developing novel therapies aimed at conditions linked to its aberrant activity, highlighting its role as a potential target in cancer treatment and other diseases. Upon activation, Abl1 undergoes autophosphorylation on specific tyrosine residues, which enhances its catalytic activity and stabilizes its active conformation. This phosphorylation event is crucial, as it allows Abl1 to effectively recognize and bind to its substrates, often involving specific sequence motifs that are enriched with tyrosine residues. Although consisting of three distinct domains, the active site is localized in the kinase domain between the C- and N-lobes where the other two domains are regulatory (Figure 1A). Several distinct structural elements have been designated to describe and facilitate the discussion of the kinase domain, including the A-loop that upon phosphorylation shifts the kinase into active conformation, the P-loop involved in ATP phosphoryl binding, and the Asp^381^–Phe^382^–Gly^383^ (DFG) sequence (numbering according to PDB ID 2G2I)^8^ where the aspartate residue adopts distinct orientations between active and inactive conformations. Once bound to its protein substrate, Abl1 catalyzes the transfer of a phosphoryl group from an ATP molecule to the hydroxyl group of tyrosine residue(s) on a target protein, resulting in a conformational switching due to the modification of the target’s net charge that shifts it into the active state.^3^ In general, the mechanism of phosphoryl transfer proceeds through a metaphosphate species either as a fully formed intermediate (dissociative mechanism) or partially bonded to the ADP and the nucleophile (associative mechanism).^9−11^ In Abl1, the tyrosine residue hydroxyl functions as a nucleophile and needs, at some point during the reaction, to be deprotonated. Proton transfer can occur either to the γ phosphoryl group or to another general base in the active site. In the case of the Abl1 enzyme, the aspartate at position 363, Asp^363^, is perfectly positioned to abstract a proton from the substrate tyrosine.^8^ Overall, the observed phosphorylation pseudo-second-order rate constant for Abl1 equals *k* = 4.4 × 10^4^ s^–1^·M^–1^ (which by Eyring equation can be approximated to activation-free energy of Δ*G*^‡^ = 11.2 kcal/mol) being on parity with other kinases at 9–12 × 10^4^ s^–1^·M^–1^, with rate enhancements of ∼10^13^ over the uncatalyzed reaction rates.^12−14^

The active site of kinases may bind one or two magnesium ions as cofactors (in addition to peptide substrate and ATP) to facilitate substrate binding and catalysis.^11,15^ All published structures of Abl1 kinase have, to date, at most a single magnesium ion bound in the active site. However, in other kinases, such as cAPK and CDK2, the presence of an additional magnesium ion has been observed.^16^ Both magnesium ions, when bound, neutralize the negative charge of the ATP substrate (Figure 1B). The Site I magnesium ion interacts directly with the charges located on the β and γ phosphoryl groups and the aspartate in the DFG segment. The second magnesium ion chelates instead to the α and γ phosphoryl groups of the ATP molecule. The lack of experimental structures of Abl1 bound to two magnesium ions leads to uncertainty about whether it utilizes both cofactors for the phosphoryl-transfer step or if the mechanism may be different from other kinases in this respect. Solving experimental structures of Abl1 with different numbers of cofactors at each reaction step has its associated challenges. This has made it difficult to understand the functional roles of the differences within the protein kinase family of enzymes.

In this study, we followed the phosphorylation reaction of Abl1 kinase, in which the enzyme transfers the γ phosphoryl group from an ATP molecule to a tyrosine residue on the peptide substrate Abltide. The starting structure for our calculations was the Abl1 kinase domain in the active conformation (PDB ID 2G2I).^8^ Conformational ensembles for the different steps of the phosphorylation reaction were generated by molecular dynamics (MD) in combination with free energy perturbation (FEP).^17^ Empirical valence bond (EVB)^18,19^ theory was subsequently used to determine free energies for the phosphorylation reaction by umbrella sampling (US).^20^ The calculations allowed us to explore different reaction mechanisms that describe Abl1 catalysis and to evaluate the effect of the ATP conformation as well as the number of magnesium ions in the active site. The best estimations of activation-free energy barriers reproduced the experimentally determined enzyme reaction rates and allowed us to propose the most probable reaction pathway, which was hitherto unknown.

## Methods

### Molecular Dynamics Simulations

The starting Abl1 kinase domain structure for the MD simulations was derived from Abl1 in the active conformation (PDB ID 2G2I).^8^ The coordinates for the missing residue atoms between Gly^250^ and Gly^253^ were taken from a structure of Abl1 with a partially activated kinase domain (PDB ID 2G2F).^8^ The ATP and peptide substrate EAIYAAPF atom coordinates were also taken from PDB entry 2G2F. The coordinates for the single Mg^2+^ were from the Abl1 structure PDB ID 2G1T.^8^ The coordinates for the ATP molecule and the two magnesium ions were based on a high-resolution structure of protein kinase A (PDB ID 1ATP).^21,22^ When necessary, the coordinate systems for the above structures were 3D transformed to match the protein PDB ID 2G2I by structurally aligning them in PyMOL by using the built-in *align* function.^23^

All MD simulations were carried out with the software package Q (version 5)^24^ utilizing the CHARMM22^25^ force field with TIP3P water; CHARMM22 was found to be suitable for QM/MM calculations employing EVB in enzyme studies.^26,27^ The enzyme system consisted of the kinase, ATP, magnesium, and a peptide substrate with a 60 Å diameter water droplet centered at the coordinates for the γ-phosphorus atom of ATP (Figure 1C). The outermost water molecules in the water sphere were restrained by radial and polarization restraints according to the SCAAS model.^28^ Protein atoms outside the water sphere were restrained to the initial coordinates with positional restraints of 200 kcal mol^–1^·Å^–2^ according to the software defaults. Charged amino acid residues close to the water boundary were neutralized to avoid unphysical forces due to insufficient screening. The system was minimized and heated by increasing the simulation time-step from 0.01 to 1 fs together with the system temperature ranging from 0.1 to 300 K over the course of 600,000 steps (510,000 steps in the system with two magnesium ions). During the equilibration phase, the solute atoms were restrained to the initial coordinates by weak positional forces. The coordinates of the final equilibrated structure were used for the calculation of the free energy profiles for the reaction.

For the simulation of the phosphorylation reaction mechanism, the EVB model was used to represent the reacting groups. This central part of the system was involved in the breaking and making of bonds, with changes in the force field parameters affecting three different charge groups consisting of 25 atoms: the α–γ phosphoryl moieties of the ATP, the tyrosine hydroxyl, and the aspartate carboxylate. The total charge of the reacting atoms was −5. For the nonreacting atoms, the charge was −3 or −1 in simulations with one or two magnesium ions, respectively. The charge of the excluded atoms, outside the water droplet, was set to zero to avoid nonphysical simulation effects. The free energy perturbation (FEP) method was used to drive the system between the states.^17^ For the EVB model, no cutoff was applied for the nonbonded interactions during the simulations. Otherwise, a 10 Å cutoff was applied for the nonbonded interactions, with the local reaction field (LRF)^29^ multipole expansion used to approximate electrostatic interactions.

The MD simulations were propagated at 1 fs time step with applied SHAKE^30^ constraints on solvent bonds and angles. Before FEP simulations were conducted, the system velocities of the equilibrated structure were randomized according to the Maxwell distribution and equilibrated for 100 ps without any positional restraints for atoms within the simulation sphere. For the dissociative mechanism, FEP simulations were carried out in 0.01 lambda increments (101 FEP windows), each 5000 fs iterations long. For the associative mechanism, 30 extra FEP lambda increments were implemented linearly beyond the lambda 0.3 step (131 FEP windows for the overall reaction). To achieve an adequate sampling of the free energy surface, each perturbation run between the EVB states consisted of more than 100 independent trajectories. The final free energy profiles were calculated by the umbrella sampling (US) method, giving a total of at least 100 different free energy profiles for each reaction step studied. The 10% lowest free energy activation barriers were used for the prediction of rate constants calculated by the Eyring equation with the transmission coefficient (κ*)* set to 100%.

The corresponding reaction in water was modeled with an ATP molecule and the tyrosine and aspartate residues. Equilibration and FEP simulations were carried out as described above for the protein system.

### The EVB Model

The kinase reaction mechanism was defined by the EVB model.^18,19^ To describe the dissociative mechanism, three EVB states were necessary: (i) the reactant state, with ATP (for all purposes here assumed to be fully charged), neutral tyrosine, and charged aspartate (atoms according to the reactive groups described), (ii) the metaphosphate state, where the γ phosphoryl group has dissociated, with tyrosine protonated and aspartate charged, and (iii) the product state in which tyrosine was deprotonated, by the aspartate functioning as a general base, and concertedly attacked the metaphosphate leading to phosphorylation. For the associative mechanism, the system was propagated directly between the reactant and product EVB states.

The trademark of the EVB method, the water reaction parametrization, was carried out after MD/FEP simulation of the corresponding reaction steps. The specific parameters of the EVB model, i.e., the gas phase energy shifts and the off-diagonal Hamiltonian matrix elements calculated by the US^20^ method, were determined to match the experimental reaction- and activation-free energies for the solution reactions. Reaction profiles for both dissociative and associative mechanisms were calculated. This requires parametrization of the metaphosphate formation as an intermediate (corresponding to the dissociative mechanism). As the reaction in water was used as a reference, the overall activation barriers in water were assumed to be of similar heights when calculating the reaction in the protein regardless of the mechanism.

Considering the experimental rate constant for tyrosine phosphorylation, the ATP^4–^ methanolysis reaction was used as a reference. The activation barrier (Δ*G*^‡^) for γ phosphoryl group transfer to methanol, calculated from a rate constant of 3.4 × 10^–10^ s^–1^·M^–1^, was equal to 30.3 kcal/mol according to the data by Wolfenden and co-workers.^14^ Since the free energy of the metaphosphate formation was assumed to be approximately at the same height as the transition state during the reaction, it was parametrized 0.5 kcal/mol below the reaction maximum. The experimental inaccessibility of the transient high-energy intermediate requires this mainly technical approximation to serve as a reference for the parametrization (adiabatic curves for the intermediate step would be at the stationary point).^31^ For the EVB model of the water reaction, a correction was made for the entropic contribution of moving the reactants from the 1 M state to the 55 M state, the contact reaction distance, which leads to a decrease in the experimental activation barrier by 2.4 to 27.9 kcal/mol. Metaphosphate formation was the rate-limiting step as the p*K*a of the alcohol attacking group in the range of 12–16 should not influence the rate according to the determined Brønstedt plot with a slope of β = 0.07.^9^ The addition of magnesium ions to the solution reaction had only negligible effects on the methanolysis of ATP^4–^ with reaction rates of 3.9 × 10^–9^ s^–1^·M^–1^ and β = 0.06.^9,14^ The parametrization of the metaphosphate intermediate formation in water gave α_0_ = −448.5 kcal/mol and off-diagonal coupling *H*_12_ = 89.3. For the GTP hydrolysis in water, an alternative derivation of the activation barrier height was carried out leading to the highest value at 29.7 kcal/mol at 1 M state (or 27 kcal/mol corrected to the 55 M).^32^ The activation barriers were of similar height and were therefore considered equivalent within the margin of experimental error.

Next, we studied the reaction as a concerted general base deprotonation of the nucleophilic tyrosine and its attack on the metaphosphate. This was expected to proceed as a thermodynamically downhill reaction, concerning the free energy. To this end, the only requirement was to determine the total free energy of the reaction. The hydrolysis of ATP in water to yield ADP and inorganic phosphate gave a reaction free energy of approximately −9 kcal/mol at a pH value equal to 7. Together with the deprotonation of the tyrosine hydroxyl by the aspartate carboxylate, the Δ*G°* for the total reaction was equal to approximately 0.6 kcal/mol based on the p*K*_a_ difference of ∼7 units for the amino acid side chain functional groups (Δ*G*^0^ = 9.6 kcal/mol). The corresponding EVB parameters for the phosphorylated tyrosine formation in the water simulation were set to α_0_ = −0.2 kcal/mol for the gas phase shifts and *H*_12_ = 125.0 kcal/mol for the off-diagonal coupling. The final protonation of the phosphate by the aspartic acid residue (general base in the previous step) before the release of the ATP was not modeled. This is a thermodynamically favorable reaction that restores the favorable equilibrium of product formation for the system.

For the associative mechanisms, the phosphorylation and deprotonation steps were carried out concertedly, allowing free mixing of the EVB states. The barrier height was the same as for the dissociative mechanism (Δ*G*^‡^ = 27.9 kcal/mol), and the reaction equilibrium was calculated to (Δ*G*^0^ = 0.6 kcal/mol). The parametrized water reaction gave α_0_ = −481.2 kcal/mol and off-diagonal coupling *H*_12_ = 65.2.

The parametrization of the water reaction, for both the associative and dissociative mechanisms, was used unchanged for the corresponding reaction simulations in the enzyme system.

## Results

We calculated activation-free energy barriers for different reaction mechanisms, conformations of the ATP, and one vs two bound magnesium ion cofactors in Abl1 kinase. This enabled the evaluation of the most probable reaction mechanism and the corresponding configuration of the active site.

### Dissociative Mechanisms

**Figure 2:**
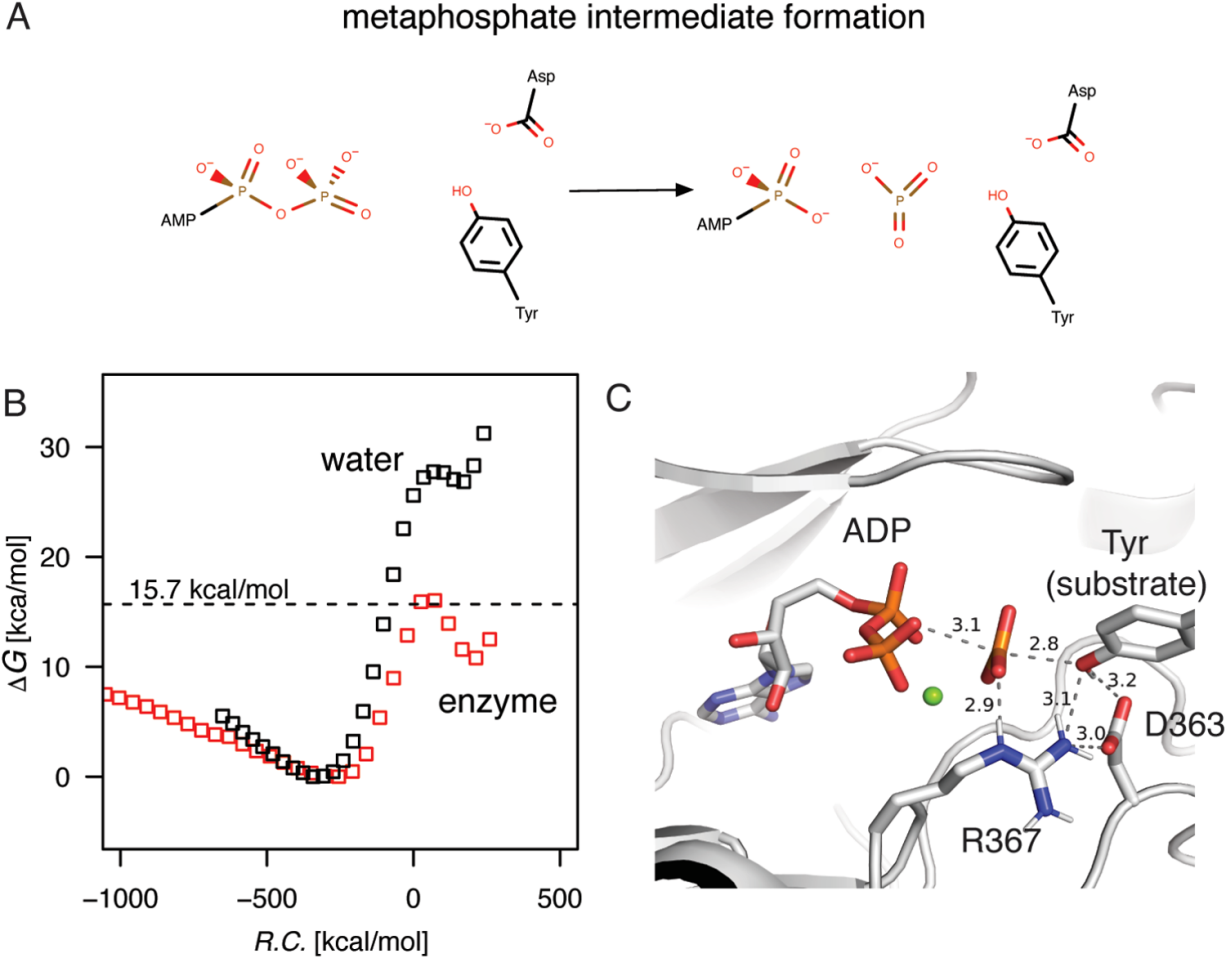
Metaphosphate formation evaluated in the water system and Abl1 enzyme. (A) The simulated reaction, where the γ phosphoryl group of ATP has dissociated to form the metaphosphate intermediate. (B) The free energy profiles for the water reaction and in the Abl1 enzyme. The average activation-free energy for the 10 lowest profiles was at 15.7 kcal/mol (marked by the dashed line), while the water reaction maximum was about 12 kcal/mol higher at 27.9 kcal/mol, indicating a transition state stabilization during the reaction. (C) In the active site, the metaphosphate species is stabilized primarily by the positive magnesium ion (the green sphere) and the hydrogen bond donating arginine residue.

The characteristic of the dissociative phosphorylation step is the completely formed planar metaphosphate located between ATP β oxygen and the tyrosine hydroxyl nucleophile (Figure 2A). The metaphosphate is described by the EVB state as an intermediate but should be very close if not identical to the transition state during the phosphorylation step. The free energy of the metaphosphate formation was parametrized, therefore, in the water reaction just below the reaction activation barrier for the uncatalyzed reaction rate of Δ*G*^‡^ = 27.9 kcal/mol. Both in water and the enzyme, at least 100 MD/FEP trajectories were calculated for the metaphosphate formation step. For the water reaction, the free energy of activation calculated by EVB/US varied little by approximately ±0.5 kcal/mol, among the trajectories. For the enzyme, there was more variability, depending on the protein configurations sampled due to the randomized starting velocities. Analysis of the 10% lowest barrier heights gave Δ*G*^‡^ = 15.7 kcal/mol with the standard error of the mean equal to 0.4 kcal/mol in the enzyme (Figure 2B). The lowest calculated activation barrier was equal to Δ*G*^‡^ = 13.9 kcal/mol. The simulation structure showed a formed metaphosphate, with the catalytic tyrosine nucleophile ready for the attack on the phosphorus atom. At the same time, the tyrosine was at a hydrogen-bonding distance from the aspartate general base (Figure 2C). The catalytic residues were arranged for the next step in the reaction, simulated as concerted tyrosine deprotonation by the aspartate and its nucleophilic attack on the metaphosphate.

**Figure 3:**
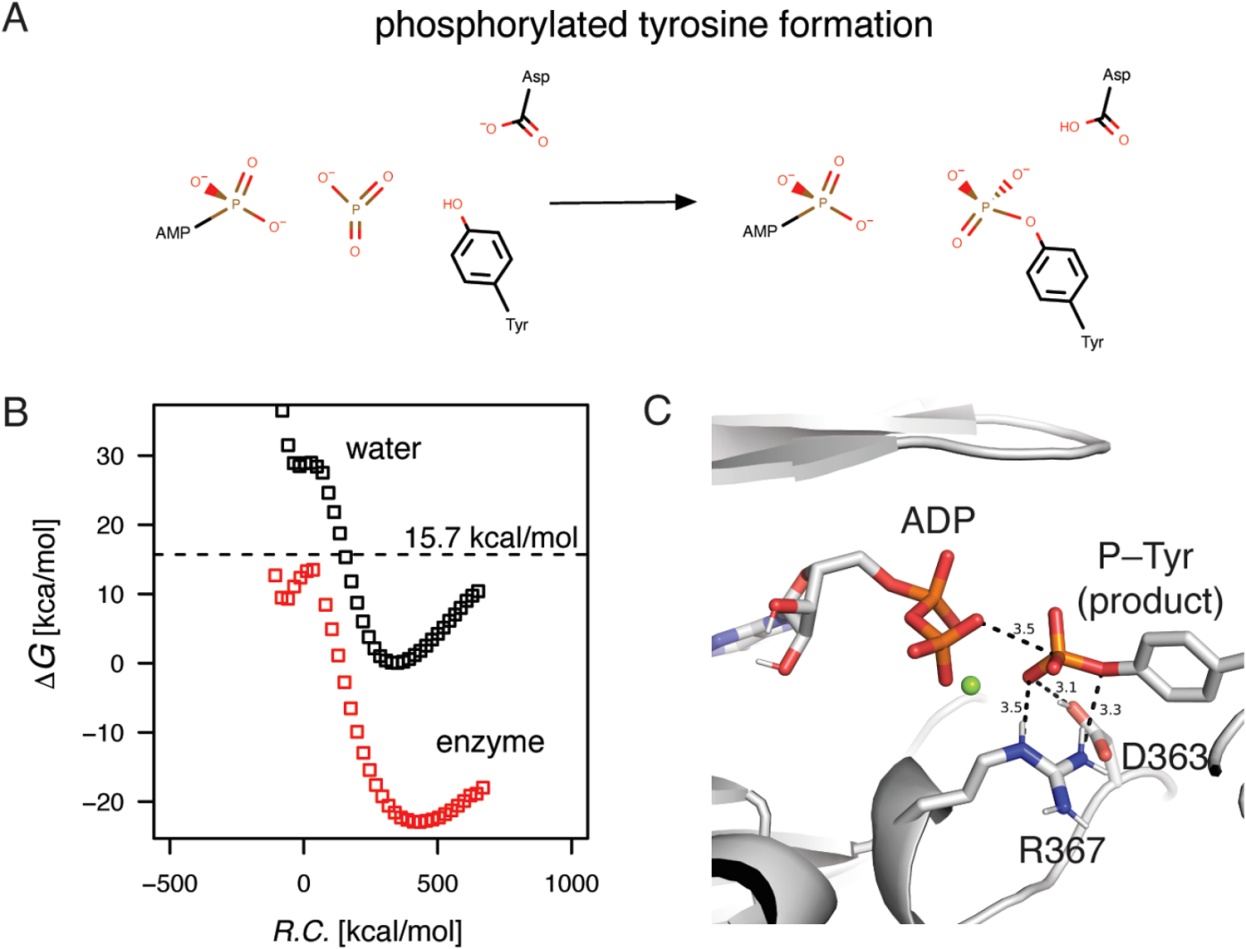
Formation of the phosphorylated tyrosine in water and the Abl1 enzyme. (A) In the final phosphorylation reaction, the metaphosphate intermediate is attacked by the tyrosine hydroxyl group, which is concertedly deprotonated by the general base aspartate. (B) The free energy profiles as determined for the phosphorylation step. For both reactions, the metaphosphate intermediate starting energy minima were shifted to match the free energy of reaction of the formation step. (C) At the end of the reaction, the phosphorylated tyrosine is hydrogen bonded to the arginine residue and stabilized by the positively charged magnesium ion.

The phosphorylation step was simulated by driving the trajectories between the formed metaphosphate state and the product state, where in the final state the tyrosine was phosphorylated and the aspartate protonated (Figure 3A). The water reaction was again parametrized at an activation barrier equal to the uncatalyzed reaction rate of Δ*G*^‡^ = 27.9 kcal/mol to match the activation barrier for the first step (Figure 3B). In the enzyme, the free energy barrier of the transition state was similar in height in comparison to the water reaction but below the maximum value for the previous reaction step. The analysis of trajectories showed, however, considerable variation for the product state in terms of end point free energies of reaction. Moreover, significant variation was observed for the water reaction where most of the minima for the product sate fall between −5 and 5 kcal/mol around the parametrized free energy of reaction value. The challenges in the calculation of phosphoryl group dissociation in the ATP have been discussed before.^33^ Most importantly, we can see that there is no dramatic increase in the activation barrier for the simulated second part of the reaction, i.e., the phosphorylation. In addition, the product state is well formed with interactions between the phosphorylated tyrosine and active site residues arginine and aspartate (Figure 3C). In the next step, proton transfer from the aspartate to the phosphorylated tyrosine was not simulated, as this would be expected to ameliorate any charge-dependent effects from the dissociation of the phosphoryl group.

### Associative Mechanism

**Figure 4:**
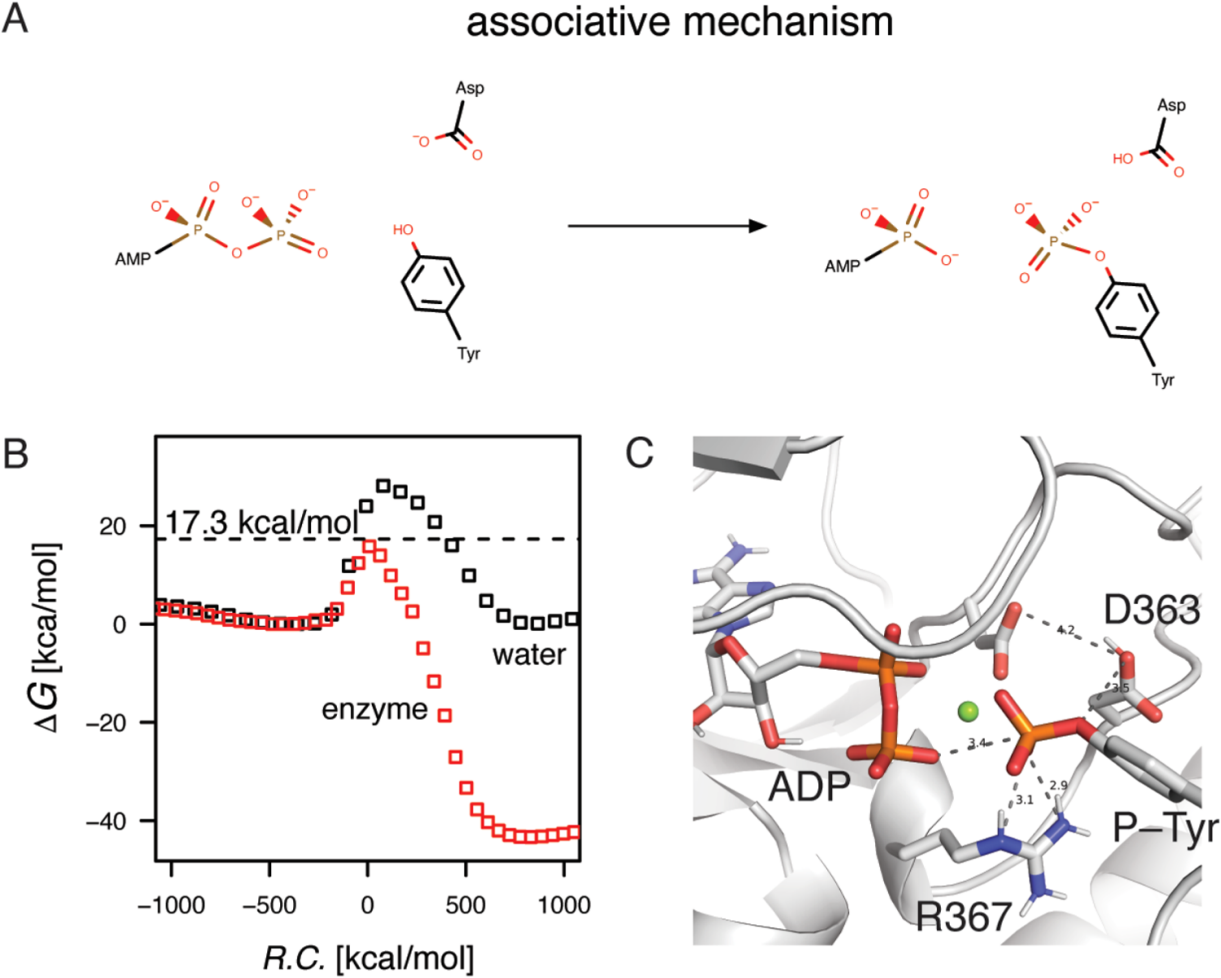
Associative mechanism of substrate phosphorylation. (A) In contrast to the dissociative mechanism, where the reaction was simulated in two steps with a formed metaphosphate intermediate, the tyrosine residue phosphorylation was modeled simultaneously as the proton transfer step. (B) The observed transition state stabilization was at 17.3 kcal/mol, in comparison to the water reaction parametrized at 27.9 kcal/mol, as previously determined for the dissociative step. (C) The formed product state was similar to the dissociative mechanism concerning the stabilization of the γ phosphoryl group by the arginine residue. Moreover, the general base lost the hydrogen bond to the tyrosine and instead was pulled toward the DFG motif aspartate.

The simulation strategy for the associative mechanism rested on letting the substrate and product states mix according to their state-dependent energies during perturbation without any intermediate steps (Figure 4A). No explicit metaphosphate was formed during the reaction as the inversion of the oxygens around the γ phosphorus atom occurred faster than the simulation time scale. The activation barrier was however higher, on average 17.3 kcal/mol for the 10 fastest reactions with a standard error of the mean equal to 0.3 kcal/mol (Figure 4B). The fastest reaction had an activation barrier of 15.8 kcal/mol. For the associative mechanism, both measures placed the energetics just about 1.5–2.0 kcal/mol above the dissociative reaction. The product state showed a well-formed phosphorylated tyrosine stabilized by the active site arginine, similar to what was observed during the dissociative mechanism (Figure 4C). The associative step, interestingly, has late proton transfer and phosphorylation steps during perturbation occurring for the substrate state at a lambda of approximately 0.2.

### Effect of Magnesium Ions on Catalysis: Two Ions vs One

**Figure 5:**
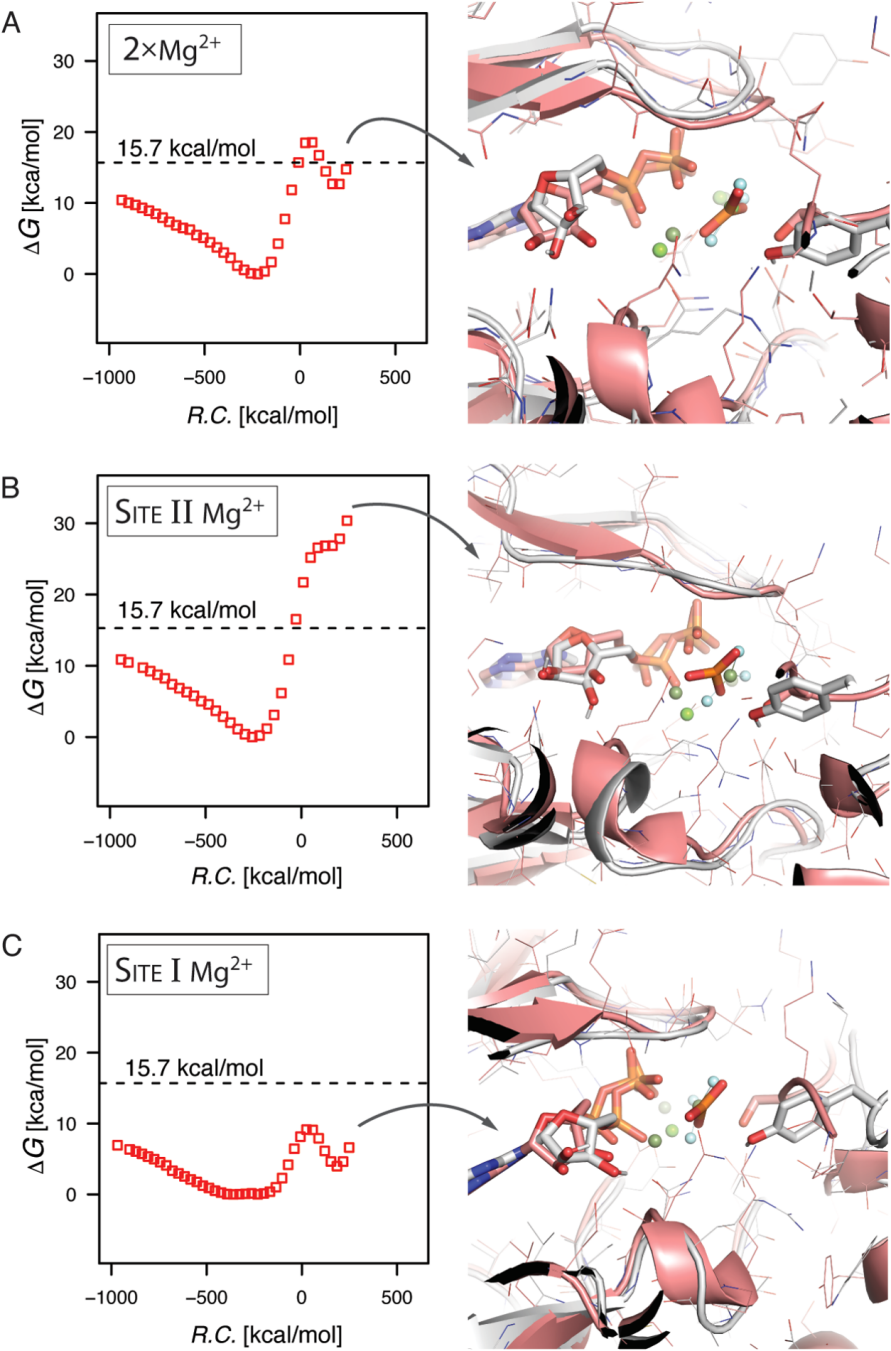
The catalytic effect of two magnesium ions compared to one in the active site of Abl1. The dissociative metaphosphate formation was simulated in the presence of two vs one magnesium ions, and the simulations were compared to the metaphosphate state found in the corresponding structure of CDK2 (PDB ID 3QHW). (A) The observed free energy barrier in the presence of both magnesium ions was higher at 18.5 kcal/mol compared with the best-observed effect with a single magnesium ion at 15.7 kcal/mol. (B) The presence of the magnesium ion in Site II (closest to the position found in PDB ID 2G1T used for the initial simulations of the dissociative mechanism) did not result in catalysis; this associated free energy barrier was observed at 26.9 kcal/mol. It was on parity with the uncatalyzed reaction in water that had a barrier height of 27.9 kcal/mol. (C) Positioning the magnesium ion in Site I resulted in the most favorable catalysis at 9.1 kcal/mol, indicating that this might be the preferred active site configuration for the reactions. In all cases, the positioning of the simulated magnesium cofactors and formed metaphosphate overlapped well with the experimental structure of the metaphosphate intermediate in CDK2.

To determine the effect of two bound magnesium ions on catalysis, the coordinates of an ATP molecule with the cofactors were taken from the structure of cAPK (PDB entry 1ATP)^21,22^ after superimposing the kinase domains. Since the fastest rate enhancement proceeded via the dissociative phosphoryl transfer, simulations were carried out for this mechanism with the FEP/EVB protocol after structure equilibration. Out of more than 100 FEP trajectories, the lowest energy barrier for the double magnesium in the active site was observed at 18.5 kcal/mol (Figure 5A). The average value of the 10 fastest catalytic rates was also much higher at 23.4 kcal/mol with a standard deviation of 2.2 (and S.E.M. equal to 0.7). The influence of the cofactors did not reach the observed catalytic effect during the associative or dissociative mechanisms. In sum, the calculations revealed that the two-ion mechanism was unproductive during Abl1 catalysis. To further evaluate the simulations, the metaphosphate state structure was overlaid on top of the corresponding structure for CDK2 with PDB ID 3QHW(16) (Figure 5A). The most noticeable difference was the 1.4 Å shift in the Abl1 bound ATP (measured at the ribose C4′) toward the phosphate-binding loop for the ATP in the CDK2 active site. The change in the ATP position was best interpreted as a too-crowded active site in the presence of two magnesium ions. The repositioning of ATP leads moreover to a less closed phosphate-binding loop and a more open active site. Simulations of the associative mechanism with two magnesium ions were on par with the dissociative mechanism.

Since the ATP conformation in other structurally determined kinases differed from the one bound in Abl1, simulations were carried out with a single magnesium ion in Site II, with ATP coordinates from PDB entry 1ATP, to evaluate the effect on catalysis. This arrangement positioned the cofactor closest to the magnesium ion coordinates found in the Abl1 structure used for the dissociative and associative mechanism simulations that were carried out initially. Nevertheless, there was still a difference of ∼1.8 Å between the bound ions in the two different kinase structures (PDB IDs 2G1T and 1ATP).^8,21,22^ Unexpectedly, the transition state was poorly stabilized in this configuration. The calculated values ended at their best at 26.9 kcal/mol despite running 100 independent trajectories (Figure 5B). Moreover, most of these had activation barriers on par with those of the water reaction without any protein or cofactor interactions contributing to the catalysis. The metaphosphate state was, as previously, well-formed with the catalytic tyrosine ready for both the nucleophilic attack and hydrogen bonding to the general base aspartate (Figure 5B).

To test the catalytic effect of the magnesium ion in Site I, we removed the coordinates for the Site II magnesium ion from the PDB ID 1ATP structure. Before the equilibration of the starting structure, this placed the ATP β phosphate and magnesium ion directly below the phosphate-binding loop. The calculations carried out for the metaphosphate state formation showed a significant stabilization with an activation barrier as low as 9.1 kcal/mol (Figure 5C). In the metaphosphate state, the magnesium ion had moved 1.7 Å from the starting coordinates, placing it now effectively one-third of the distance toward the Site II magnesium (Figure 5C). The displacement was partially due to the repositioning of the γ phosphoryl group before the metaphosphate state formation and the following attack by the tyrosine residue hydroxyl.

## Discussion

Kinases, including Abl1, are known to accelerate the phosphorylation of proteins more than ∼10^13^ over the uncatalyzed reaction rates.^12,13^ We have investigated what reaction mechanism can realize the experimentally observed catalytic rates by exploring different reaction pathways, evaluating distinct conformations of the substrate ATP, and determining the influence of magnesium ion cofactors. The starting point for the calculations was the active state conformation of the enzyme, with the secondary structure elements orientated toward catalytically favorable conformations. For example, the DFG segment was flipped so that the aspartate instead of phenylalanine pointed toward the substrates. Besides comparing the calculated free energy barriers with the experimental rates, a comparison was also made between the modeled metaphosphate reaction intermediate and the one observed in CDK2/Cyclin A kinase bound to ADP and MgF_3_^–^ (PDB ID 3QHW). Although Abl1 and CDK2 are distinct kinases with different substrates (Tyr vs Ser/Thr, respectively), the catalyzed reaction is highly similar in both cases which allowed us to carry out a structural comparison of their active sites.

The simulation of the reaction mechanisms in Abl1 started in the structure bound to substrate-mimicking ATP analog–peptide conjugate.^8^ From here, the substrates ATP and the tyrosine-containing peptide were built with the addition of the magnesium ion cofactor. Although the γ phosphate was positioned just outside the phosphate-binding P-loop, we carried out simulations to calculate the activation barrier for the phosphoryl-transfer reaction in this Abl1 structure as a reference for mechanistic studies. Out of the two choices for catalysis, the dissociative pathway following a fully formed metaphosphate intermediate preferentially showed better stabilization of the transition state. The best-observed catalytic rate with Δ*G*^‡^ = 13.9 kcal/mol was slower than the experimentally measured values of Δ*G*^‡^ = 11.2 kcal/mol. The presence of aspartate in the Abl1 active site at the reactive distance from the substrate tyrosine made the general base-assisted mechanism straightforward. We simulated the tyrosine attack on the metaphosphate intermediate and general base deprotonation concertedly because the experimental data demonstrated a flat Brønstedt plot. In some enzyme cases, when there is no clear option for a general base, nucleotide hydrolysis may proceed via a substrate-assisted mechanism. Computational simulations of GTP hydrolysis, as well as phosphatase reaction mechanism, demonstrated such a mechanism of catalysis.^31,32,34,35^ In both cases, a proton is abstracted from a water molecule or a general acid cysteine by the γ phosphoryl group, where associative hydrolysis leads to product formation. The presence of the general base in Abl1 makes the substrate-assisted mechanism less likely.

Although the predicted and measured rates for the dissociative mechanism differed by only approximately 3 kcal/mol, it was worth investigating a couple of possibilities that may explain the discrepancy not directly falling within the measurement error. To evaluate the influence of an alternative conformation of the ATP phosphoryl groups and the effect of magnesium ions in the active site, simulations were based on their placement as determined in the structure of the cAPK enzyme.^21,22^ We carried out simulations with two magnesium ions, but also evaluated their individual effects by starting additional independent simulations with only a single bound ion at the time. The best catalysis resulted in an activation barrier of 9.1 kcal/mol observed with the magnesium ion in Site I, directly below the P-loop and the γ phosphoryl group and above DFG motif aspartate. The presence of both magnesium ions in the active site led to the metaphosphate intermediate almost overlapping the experimentally determined metaphosphate atom positions (in the CDK2 kinase structure),^16^ however, no significant catalytic effect was observed relative to the water reaction. With two magnesium ions, the formation of the enzyme–ATP substrate complex may be favorable, but the active site cannot follow the charge transfer during the reaction leading to the unfavorable reorganization energy. The calculations also demonstrated that placing positive charges in the vicinity of the γ phosphoryl group does not lead to transition state stabilization. The cofactor must be positioned in an orientation favorable for catalysis. In the case of CDK2 enzyme, the calculations indicate that the more favorable mechanism may require both magnesium ions present,^36^ however, a direct comparison with experimentally determined rates with high magnesium ion concentrations is challenging as these may influence both substrate binding and catalysis.^16^

This study indicates that Abl1’s reaction involves a single Mg^2+^ ion, but it does not unequivocally refute the possibility of other configurations of the protein that may catalyze with two Mg^2+^ ions. Since no crystal structure of Abl1 exists with ATP/2Mg^2+^, we modeled the structure with two Mg^2+^ ions based on the structure of a different kinase, cAPK. While the proteins are structurally similar and Abl1 was energy minimized before the simulations, the structure of the protein with two magnesium ions might adopt other conformations that are more prone to catalysis but which we could not model. We see this as a necessary limitation. In principle, it should be possible to model different complex conformations using MD simulations; however, Mg^2+^ ions are highly polarizing, and force field parameters may be insufficient for accurate modeling of the active site with two such ions. Alternatively, carrying out simulations with QM/MM would be instead limited in sampling power. Considering other factors that might influence the discrepancy between the experiment and calculations, it should be reminded that the measurements are carried out in a solution containing a certain concentration of substrate, buffers, and other components that might affect the reaction rate somewhat but cannot be modeled accurately in the simulations. In addition, the choice of the force field and the coupling to the EVB might also lead to some deviation in the calculated barriers. Nevertheless, the relative values for the activation-free energy should be representative of the different mechanisms.

## Conclusions

Despite the similarity in sequence and structure, this study shows that catalysis of phosphate transfer from ATP to a peptide substrate might proceed differently when Abl1 is compared to other kinases. It was shown that a dissociative mechanism dominates, with a metaphosphate intermediate formed before the phosphorylation of the substrate tyrosine and the proton transfer step. This knowledge might be utilized for enzyme design and structure-based drug design. Specifically, type-I kinase inhibitors, which bind to the active site, are often less prone to resistance due to mutations in the active site but are perceived as less specific. Identification of unique catalytic divergences may allow the design of more specific inhibitors, taking advantage of the preference for *lock and key* mechanism of such inhibitors.^37^

## Abbreviations

Abl1: Abelson kinase 1
CML: chronic myeloid leukemia
EVB: empirical valence bond
FEP: free energy perturbation
US: umbrella sampling
